# The observed difference of macrophage phenotype on different surface roughness of mineralized collagen

**DOI:** 10.1093/rb/rbz053

**Published:** 2020-01-25

**Authors:** Jun Li, Yu-Jue Zhang, Zhao-Yong Lv, Kun Liu, Chun-Xiu Meng, Bo Zou, Ke-Yi Li, Feng-Zhen Liu, Bin Zhang

**Affiliations:** 1 Department of Oral and Maxillofacial Surgery, School and Hospital of Stomatology, Shandong University & Shandong Provincial Key Laboratory of Oral Tissue Regeneration & Shandong Engineering Laboratory for Dental Materials and Oral Tissue Regeneration, Jinan, Shandong 250012, People’s Republic of China; 2 Liaocheng People’s Hospital, Medical College of Liaocheng University, Liaocheng 252000, People’s Republic of China; 3 Department of Materials Science and Engineering, Tsinghua University, Beijing 100084, People’s Republic of China

**Keywords:** inflammatory response, mineralized collagen, roughness, macrophage, polarization

## Abstract

Biomaterials regulate macrophages and promote regeneration function, which is a new hot pot in tissue engineering and regenerative medicine. The research based on macrophage materials biology has appeared happy future, but related research on regulating macrophages and promoting tissue regeneration is still in its infancy. The surface roughness of biomaterials is one of the important factors affecting macrophage behavior. Previous study also found that the surface roughness of many biomaterials regulating macrophage polarization, but not including mineralized collagen (MC). In this study, we designed and fabricated MC with different roughness and investigated the influence of MC with different roughness on macrophages. In the study, we found that on the rough surface of MC, macrophages exhibited M1 phenotype-amoeboid morphology and high-level secretory of inflammatory factor (tumor necrosis factor-α and interleukin-6), while smoother surface exhibited M2 phenotype. These data will be beneficial to understand the mechanism deeply and enrich biomaterials tissue regeneration theory, provide a new train of thought biomaterials inducing tissue regeneration and repair and guide the optimum design of new biomaterials, development and reasonable applications.

## Introduction

**Figure 1:**
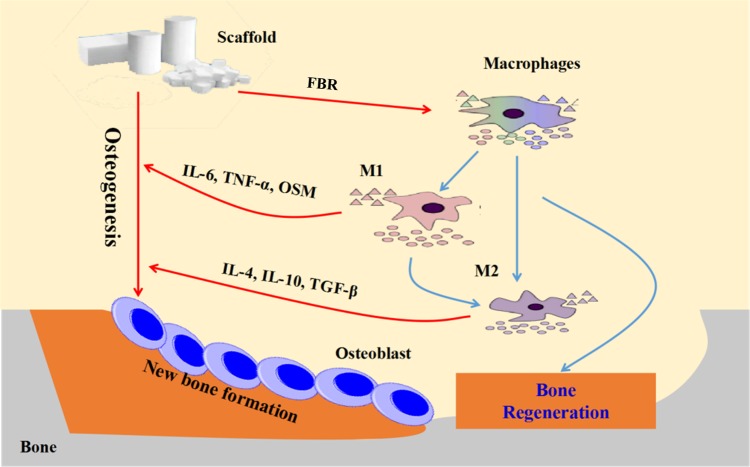
Schematic diagram of macrophages interactions in bone regeneration

The immune system plays an important role in the success of tissue engineering strategies. In the recent, more and more experts and scholars have become increasingly recognized the importance of local environment surrounding regenerating tissue, and turned much attention to how the immune system can be manipulated to achieve the purpose of implanted scaffolds, especially in inhibiting foreign body rejection and strengthening the binding of scaffolds to natural tissue [1]. When implanted into human body, all biomaterials may trigger a series of host responses. Macrophages are the main cell types in acute and chronic inflammation, as well as the following wound healing or fibrosis reactions [2]. Under different conditions, macrophages can be differently polarized, exhibit M1-(proinflammation) and M2-(tissue repair) phenotypes and secrete different cytokines and small molecules [3]. The schematic diagram of macrophages interactions in bone regeneration was shown in Fig. 1. It is known that the transition and balance between M1- and M2-phenotype are essential for tissue repair. The disorder of macrophage polarization and failure to restore M1 and M2 phenotypes to a normal balance may lead to the formation of foreign body giant cells, which is related to the failure of implant materials [4]. After implantation, a series of events caused by the interaction between immune cells and biomaterials surface properties determine the biomaterials fate [5–7].

Previous study also found that the influence of the surface roughness of biomaterial implantations on bone resorption. Rae and his colleagues [8] found that PTFE, epoxy resin and HDP materials with rough surfaces to which macrophages adheration had been shown to influence the amount of more bone resorption that they stimulated than did those with a smooth surface (PMMA). Olivares-Navarrete and his colleagues [9] found that the activation of inflammatory macrophages (M1-like) induced by smooth titanium and the activation of macrophages induced by hydrophilic rough titanium were similar to those induced by anti-inflammatory M2-like state. But till now, no literature was reported about different surface roughness of mineralized collagen (MC)-regulated macrophage polarization to influence bone regeneration.

MC is the basic building block of hierarchically organized structures of natural bone [10]. MC was prepared *in vitro* biomimetic mineralization and had been shown definite advantage in degradation rapid, stiffness and promoting osteogenic differentiation of human mesenchymal stem cells [11, 12]. Cui and his colleagues [13] had shown that the MC group was more likely to induce macrophage differentiation into M2 than the HA group. Whether the surface topography of MC will affect the polarization and function of macrophages has not been reported.

In this study, we induced THP-1 cells with PMA into macrophages and examined the morphology and cytokine secretion of macrophages cultured on MC with different surface roughness.

## Materials and methods

### Preparation and characterization of MC with different surface roughness

#### Preparation of MC with different surface roughness

The MC membrane was produced by Beijing Allgens Medical Science and Technology Co., Ltd. MC membrane with different surface roughness was prepared by following two main steps.

In the first step, the biomimetic MC was prepared via an *in vitro* biomimetic mineralization process [14]. Water solutions containing Ca^2+^ or ${\text{PO}}_{4}^{3-}$ were added into an acidic collagen solution, respectively; then the pH value and temperature of the liquid mixture were adjusted to form MC deposition. This step was similar to the biomineralization process of the natural bone. During such process, the nucleation and growth of HA crystals were directed by the collagen fibrils. The deposition was washed three times, and then freeze-dried to obtain porous MC with different surface roughness according to the amount of water used for washing.

**Figure 2:**
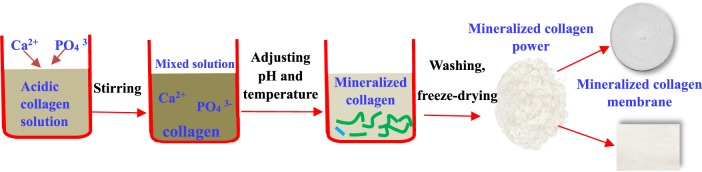
The product process of MC membrane

In the second step, after thorough freeze-drying, the MC membrane was finally molded by rolling. The final MC membrane of different surface roughness was sterilized by ^60^Co radiation. They were divided into four groups: MC-A, MC-B, MC-C and MC-D. The MC used in this study was hierarchical self-assembled nano-hydroxyapatite/collagen composites, which was synthesized via biomimetic mineralization process *in vitro* as previous reported (as shown in Fig. 2).

#### Characterization of MC membrane with different surface roughness

The surface roughness was examined using a field emission scanning electron microscope (FESEM; SU8000) at 6 kV and a China Roughness Measuring Instrument (Model 2206).

### Cell culture on MC membrane with different surface roughness

THP-1 cell line was presented by Liaocheng People’s Hospital Oral Biomaterial Laboratory and cultured in 1640 RPMI (Gibco by Life Technologies) supplemented with 10% fetal bovine serum (Gibco), penicillin (100 μg/ml) and streptomycin (100 μg/ml) (Sangon Biotech) at 37°C in a humidified atmosphere of 5% CO_2_. Cells were scraped, counted and diluted to 1 × 10^5^ cells/ml when they achieved ∼80–90% confluence. Before cultured on the materials, 100 ng/ml PMA (Sigma) was used to induce them into macrophages. As CD14 is a macrophage surface marker, we used CD14 antibody (APC anti-human CD14, Biolegend) to detect whether it successfully differentiated into macrophages. Then macrophages were cultured on the surface of different MC membrane. The MC membrane was placed in 24-well plate, incubated in phosphate-buffered saline (PBS; Sangon Biotech) medium for each 4 h prior to macrophage seeding. 1 ml cell suspension was added on each substrate.

### Morphology and micromorphology of macrophage cultured on different MC membrane

The 1 × 10^6^ monocytes were seeded on the surface of each well, cultured for 1 and 3 days, respectively, and the macrophages were rinsed with PBS and fixed with 2.5% glutaraldehyde (Sangon Biotech) aqueous solution for 20 min. After rinsing for three times, they were dehydrated by 30, 50, 70, 90 and 100% gradient ethanol (Sangon Biotech) solution, each concentration was 10 min ×2 times, then placed in a freeze dryer, lyophilized 1 h and slowly sealed back to temperature. Then gold (platinum) was sprayed on the surface of the sample for 5 min using an ion sputtering apparatus to improve surface conductivity. After the sample preparation was completed, using a FESEM (SU8000) to observe the population morphology at a low magnification, and the individual morphology at a high magnification.

### Acridine orange/propidium iodide double staining morphological analysis

Approximately 2 × 10^5^ cells/ml of macrophages were seeded on the surface of MC membrane and incubated for 24 h at 37°C in a humidified, 5% CO_2_ atmosphere. After the cells were incubated, the cells were washed with PBS. The cell suspension (10 μl) was placed on the glass slide and mixed with 10h μl of acridine orange (AO; 50 μg/ml) and propidium iodide (PI; 50 μg/ml). The cells were observed under a fluorescence microscope (Leica, Germany).

### Cytokine determinations

M1-cytokine (tumor necrosis factor-α [TNF-α] and interleukin [IL]-6) and M2-cytokine (IL-10 and IL-4) levels were measured with sandwich enzyme-linked immunosorbent assays as prescribed by the manufacturer (Abcam).

### Statistical analysis

All the analyses were performed using the software SPSS 22.0 (IBM SPSS, Armonk, NY, USA). All the data were expressed as means ± SD and analyzed using one-way ANOVA followed by least significant difference *post hoc* test. A level of significance was set at *P* < 0.05.

## Results

### Characterization of different MC membrane

**Figure 3:**
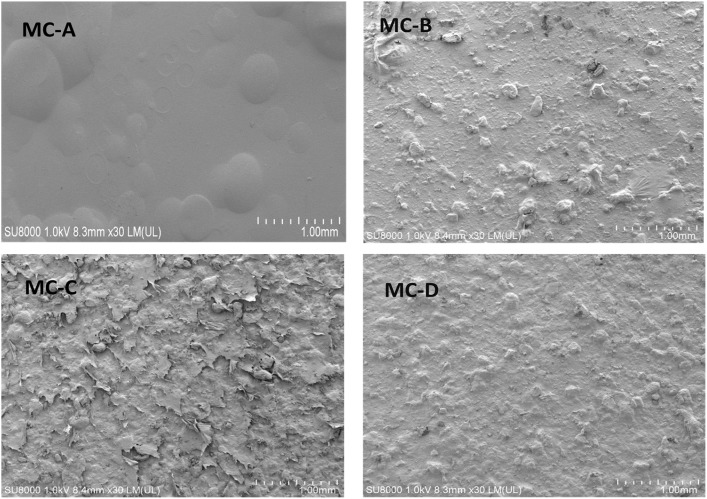
FESEM micrographs of MC-A, MC-B, MC-C and MC-D

The Ra, average roughness and value of the replica surfaces of MC membrane were shown in Table 1. The surface roughness of different MC membrane had been extensively characterized. FESEM micrographs of the samples were shown in Fig. 3.

**Table 1:** The difference roughness of different materials

Materials	MC-A	MC-B	MC-C	MC-D
Roughness (µm)	0.92 ± 0.05	6.41 ± 0.15	8.31 ± 0.21	12 ± 0.36

### Identification of macrophages

**Figure 4:**
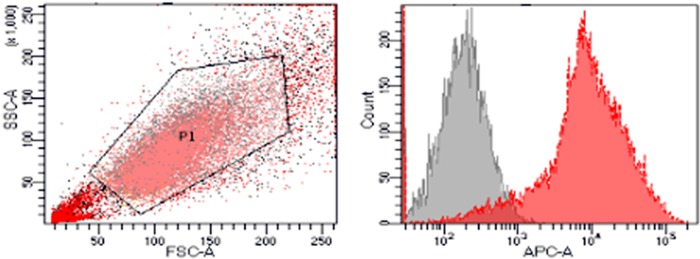
Flow cytometry detection of macrophage surface marker CD14

Due to the high expression of CD14 on the surface of macrophages, CD14 was selected as a macrophage surface marker and its purity was detected by flow cytometry. The results of flow cytometry were shown in Fig. 4. The purity of macrophages was >97%.

### Population and individual morphology of macrophages grown on different MC membrane

**Figure 5:**
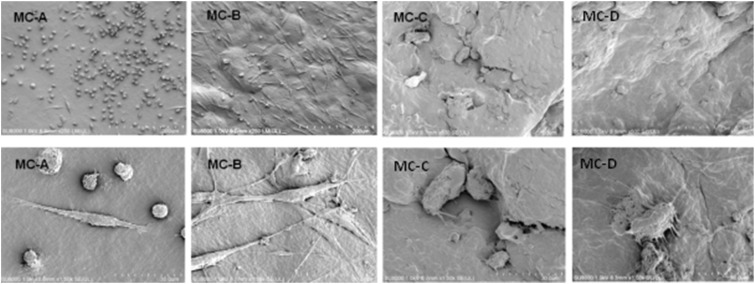
The population and individual morphology of macrophages cultured on different groups of 1 day. The upper row showed the population morphology and the lower row showed the individual morphology

The results of the number and morphology of macrophages on different materials after cultured on the surface of each group for 1 day were shown in Fig. 5. From Fig. 5, we found that the macrophages displayed dramatically different shapes and quantities on the different surface roughness of MC membrane. When compared with MC-C and MC-D groups, there were more macropahges on MC-A and MC-B groups. Most macrophages on the MC-A group had a round shape and MC-B groups exhibited fusiform or spindle shape, while the macrophages on groups MC-C and MC-D protruded many protrusions.

### Cell activity cultured on different MC membrane *in vitro*

**Figure 6:**
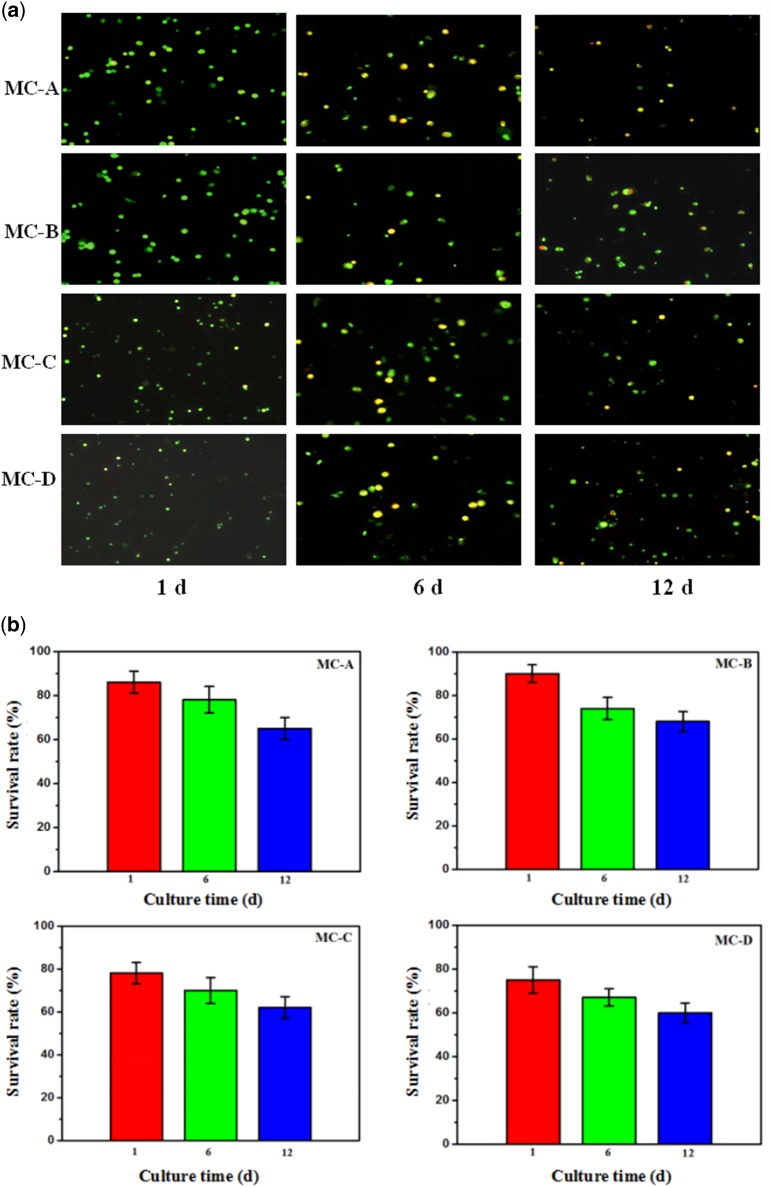
**(a)** The AO/PI staining results about cell activity *in vitro*, **(b)** cell survival rates under fluorescence microscope (no significant difference among the four groups after 12 days, *P* < 0.01)

When cells were stained with AO/PI, the dead cells produced a classical orange to red fluorescence while live cells showed a green glow, the results were shown in Fig. 6a. These results indicated that the survival rates of MC-C and MC-D groups were lower than MC-A and MC-B groups after 1 day; however, there was no significant difference between the four groups after 12 days. From Fig. 6b, the results showed the survival rate of cells can still remain above 60%.

### Cytokine release

The release of cytokines was influenced by the surface roughness of MC membrane on which macrophages were cultured.

#### Secretion of M1-cytokines

**Figure 7:**
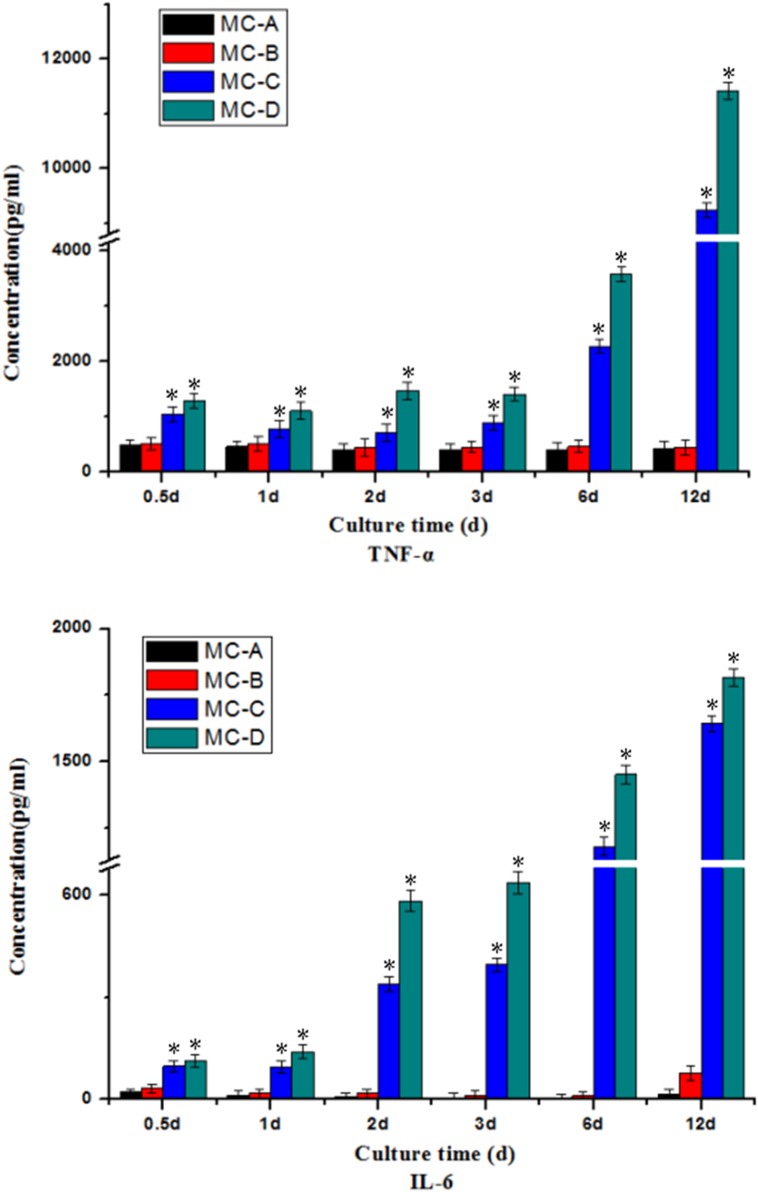
Secretion of M1-(proinflammation) cytokines: TNF-α and IL-6. *Significant difference (*P* < 0.05) compared with the MC-A group

The secretion level of proinflammation cytokines was different in each group with the prolongation of time (as shown in Fig. 7). After 3 days, the secretion of TNF-α and IL-6 by macrophages cultured on the MC-C and MC-D groups increased significantly compared with MC-A and MC-B.

#### Secretion of M2-cytokines

**Figure 8:**
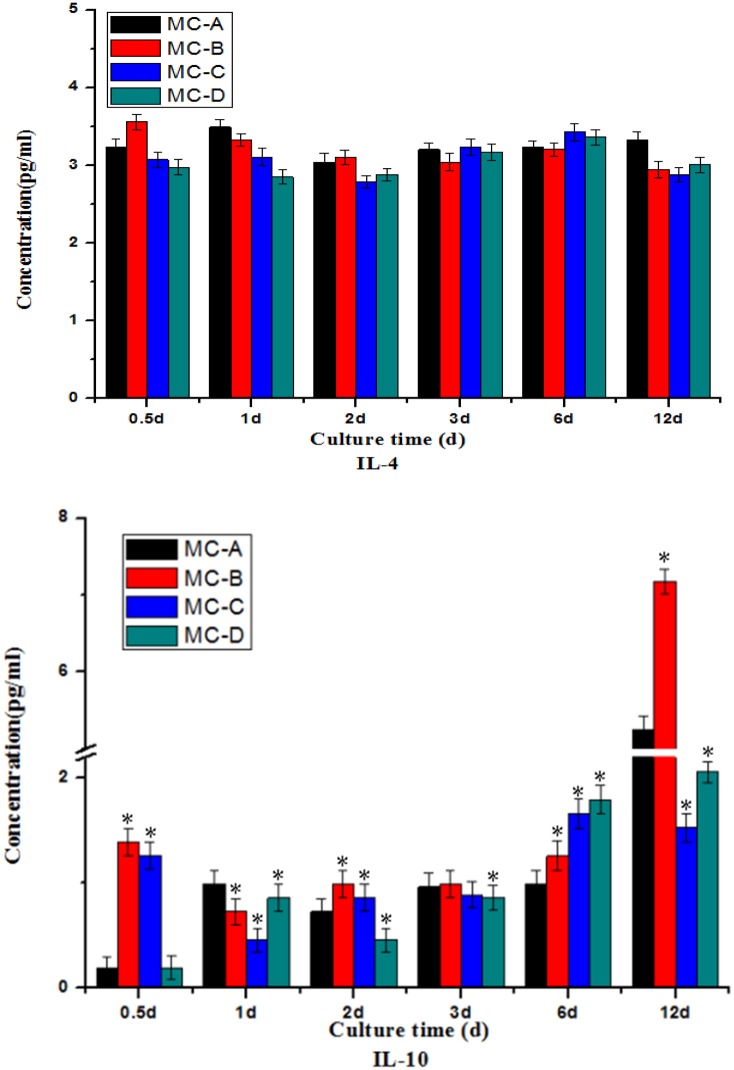
Secretion of M2-(tissue repair) cytokines: IL-4 and IL-10. *Significant difference (*P* < 0.05) compared with the MC-A group

In terms of the amount of secretion, the IL-4 concentration in the MC-A group was higher, but there was no statistically significant difference (as shown in Fig. 8). After 6 days, the secretion of IL-10 increased in MC-A and MC-B groups, whereas MC-C and MC-D showed no significant changes.

## Discussion

As multifunctional immune cells, macrophages play a key role in tissue regeneration and inflammation because of their various phenotypes and plasticity [15]. Macrophages respond to biomaterials by ‘re-educated’ and are polarized into M1 or M2 phenotype. The M1 macrophages are characterized by production of proinflammatory cytokines including TNF-α, IL-1β and IL-6, and at the beginning of the inflammatory response, the host immune status plays a role, but also leads to inflammatory lesions in the normal tissues of the body. Whereas M2 macrophages are commonly associated with secretion of IL-4, IL-10 and IL-13 and play an important part in the late stage of inflammation [16, 17]. Therefore, macrophages play a key role in bone development and regeneration and the different ratios of M1 and M2 can indicate the direction of inflammation, healing and reconstruction [18].

The morphological changes of biomaterials caused by surface topography are closely related to macrophage function and polarization [19]. Roughness can directly affect cell adhesion and spreading through its effects on protein adsorption [20, 21]. It is still controversial about the influence of surface roughness on macrophage polarization. McWhorter *et al*. [22] have shown that M1 macrophages exhibited round, pancake-like shape, which have bigger cell surface area than unstimulated M0 macrophages, whereas M2 macrophages have elongated morphology. Barth *et al*. [23] found that the secretion of cytokines on roughness surface was closer to the activity of M2-like phenotype. Although others found that unstimulated macrophages increased the secretion of the proinflammatory cytokine (TNF-α) when surfaces became rough [24]. This study indicated that surface roughness of MC affected the adhesion and diffusion of macrophages, and can also regulate the release of inflammatory cytokines and chemokines from macrophages. The shape changes of macrophages under the stimulation of different surface roughness strongly imply a possible modulation of the immune environment.

It comes to light that the balance of M1/M2 macrophages should influence tissue regeneration and wound healing after biomaterials implanted into the body [13]. Many efforts need to determine the appropriate early immune environments to activate osteogenesis-enhancing M2 macrophages and enhance new bone regeneration. Prolonged M1 polarization could increase the release of fibrosis cytokines from M2 macrophages, and it resulted in fibrocapsule formed. As a comparison, a timely and effective transformation of M1 macrophage phenotype could lead to the enhanced cytokine release pattern of M2 macrophage osteogenesis, and then formed a new bone tissue [25]. Our study suggested that the increase on surface roughness of MC enhanced the secretion of proinflammatory cytokines from macrophages and the increase on surface roughness of MC could induce macrophages polarization toward M1 than M2 evidently. Yang *et al*. [19] found that the knockout of IL-6 delayed the maturity, mineralization, and remodeling of callus, indicating that IL-6 played the essential role in the early stage of fracture healing. Xiao and his colleagues [26, 27] also had shown that the generated osteoimmune environment subsequently affected the osteogenic differentiation of BMSCs and the new bone formation. It can be beneficial that in the early stages of fracture repair, inflammation can also lead to new bone formation. This research indicated that the osteoimmunomodulation could be manipulated through biomaterials physicochemical properties, which implied a valuable strategy to develop advanced bone biomaterials with favorable osteoimmunomodulatory properties.

Therefore, the induction of macrophage polarization by biomaterials to promote bone regeneration has become a new direction in the design of bone repair biomaterials. Much research should be required a better and deeper understanding of macrophage switching patterns and the mechanism of that affected the bone healing process. This understanding will lead to the rapid development of ‘smart' bone substitute biomaterials with immunomodulatory function.

## Conclusion

This study demonstrated that MC surface topography, particularly the surface roughness, modulated the population and individual morphology as well as the production of cytokines such as TNF-α, IL-6, IL-4 and IL-10 from the macrophage cell line extracted in a time-dependent manner. These findings provide a basis for modulating macrophage polarization by MC surface optimal design to regulate host response. And we need further research to obtain more details on the biomaterial-dependent reaction of macrophages at the molecular level, as well as the role of immunomodulatory properties of bone repair materials in bone regeneration and repair.

## References

[1] Molina ER, Smith BT, Shah SR et al Immunomodulatory properties of stem cells and bioactive molecules for tissue engineering. J Control Release 2015;219:107–18. 26307349 10.1016/j.jconrel.2015.08.038

[2] Zhou G, Groth T. Host responses to biomaterials and anti-inflammatory design-a brief review. Macromol Biosci 2018;18:1800112. 10.1002/mabi.201800112 29920937

[3] Dong L, Wang C. Harnessing the power of macrophages/monocytes for enhanced bone tissue engineering. Trends Biotechnol 2013;31:342–6. 23623371 10.1016/j.tibtech.2013.04.001

[4] Fearing BV, Van Dyke ME. In vitro response of macrophage polarization to a keratin biomaterial. Acta Biomater 2014;10:3136–44. 24726958 10.1016/j.actbio.2014.04.003

[5] Mavrogenis AF, Dimitriou R, Parvizi J et al Biology of implant osseointegration. J Musculoskelet Neuronal Interact 2009;9:61–71. 19516081

[6] Anderson JM, Rodriguez A, Chang DT. Foreign body reaction to biomaterials. Semin Immunol 2008;20:86–100. 18162407 10.1016/j.smim.2007.11.004 PMC2327202

[7] Novak ML, Koh TJ. Macrophage phenotypes during tissue repair. J Leukoc Biol 2013;93:875–81. 23505314 10.1189/jlb.1012512 PMC3656331

[8] Murray DW, Rae T, Rushton N. The influence of the surface energy and roughness of implants on bone resorption. J Bone Joint Surg 1989;71:632–7. 10.1302/0301-620X.71B4.2670951 2670951

[9] Hotchkiss KM, Reddy GB, Hyzy SL et al Titanium surface characteristics, including topography and wettability, alter macrophage activation. Acta Biomater 2016;31:425–34. 26675126 10.1016/j.actbio.2015.12.003 PMC4728000

[10] Quade M, Schumacher M, Bernhardt A et al Strontium-modification of porous scaffolds from mineralized collagen for potential use in bone defect therapy. Mater Sci Eng 2018;84:159–67. 10.1016/j.msec.2017.11.038 29519425

[11] Wang Y, Hua Y, Zhang Q et al Using biomimetically mineralized collagen membranes with different surface stiffness to guide regeneration of bone defects. J Tissue Eng Regen Med 2018;12:1545–55. 29691999 10.1002/term.2670

[12] Liu Y, Liu S, Luo D et al Hierarchically staggered nanostructure of mineralized collagen as a bone-grafting scaffold. Adv Mater 2016;28:8740–8. 27530607 10.1002/adma.201602628

[13] Shi XD, Chen LW, Li SW et al The observed difference of RAW264.7 macrophage phenotype on mineralized collagen and hydroxyapatite. Biomed Mater 2018;13:041001. 29516867 10.1088/1748-605X/aab523

[14] Liao SS, Cui FZ, Zhang W et al Hierarchically biomimetic bone scaffold materials: nano-HA/collagen/PLA composite. J Biomed Mater Res 2004;69:158–65. 10.1002/jbm.b.20035 15116405

[15] Quan H, Kim Y, Chul Park H et al Effects of phosphatidylserine-containing supported lipid bilayers on the polarization of macrophages. J Biomed Mater Res 2018;106:2625–33. 10.1002/jbm.a.36454 29781181

[16] Ben-Mordechai T, Holbova R, Landa-Rouben N et al Macrophage subpopulations are essential for infarct repair with and without stem cell therapy. J Am Coll Cardiol 2013;62:1890–901. 23973704 10.1016/j.jacc.2013.07.057

[17] Wynn TA, Vannella KM. Macrophages in tissue repair, regeneration, and fibrosis. Immunity 2016;44:450–62. 26982353 10.1016/j.immuni.2016.02.015 PMC4794754

[18] Vi L, Baht GS, Whetstone H et al Macrophages promote osteoblastic differentiation in-vivo: implications in fracture repair and bone homeostasis. J Bone Miner Res 2015;30:1090–102. 25487241 10.1002/jbmr.2422

[19] Yang X, Ricciardi BF, Hernandez-Soria A et al Callus mineralization and maturation are delayed during fracture healing in interleukin-6 knockout mice. Bone 2007;41:928–36. 17921078 10.1016/j.bone.2007.07.022 PMC2673922

[20] Rostam HM, Singh S, Vrana NE et al Impact of surface chemistry and topography on the function of antigen presenting cells. Biomater Sci 2015;3:424–41. 26222286 10.1039/c4bm00375f

[21] Ma QL, Zhao LZ, Liu RR et al Improved implant osseointegration of a nanostructured titanium surface via mediation of macrophage polarization. Biomaterials 2014;35:9853–67. 25201737 10.1016/j.biomaterials.2014.08.025

[22] McWhorter FY, Wang T, Nguyen P et al Modulation of macrophage phenotype by cell shape. Proc Natl Acad Sci USA 2013;110:17253–8. 24101477 10.1073/pnas.1308887110 PMC3808615

[23] Barth KA, Waterfield JD, Brunette DM. The effect of surface roughness on RAW 264.7 macrophage phenotype. J Biomed Mater Res 2013;101:2679–88. 10.1002/jbm.a.34562 23427077

[24] Refai AK, Textor M, Brunette DM et al Effect of titanium surface topography on macrophage activation and secretion of proinflammatory cytokines and chemokines. J Biomed Mater Res 2004;70:194–205. 10.1002/jbm.a.30075 15227664

[25] Chen Z, Klein T, Murray RZ et al Osteoimmunomodulation for the development of advanced bone biomaterials. Mater Today 2016;19:304–21.

[26] Chen Z, Bachhuka A, Han S et al Tuning chemistry and topography of nanoengineered surfaces to manipulate immune response for bone regeneration applications. ACS Nano 2017;11:4494–506. 28414902 10.1021/acsnano.6b07808

[27] Chen Z, Wu C, Gu W et al Osteogenic differentiation of bone marrow MSCs by b -tricalcium phosphate stimulating macrophages via BMP2 signalling pathway. Biomaterials 2014;35:1507–18. 24268199 10.1016/j.biomaterials.2013.11.014

